# Culture of Blame—An Ongoing Burden for Doctors and Patient Safety

**DOI:** 10.3390/ijerph16234826

**Published:** 2019-12-01

**Authors:** Ognjen Brborović, Hana Brborović, Iskra Alexandra Nola, Milan Milošević

**Affiliations:** 1 University of Zagreb, School of Medicine, Andrija Štampar School of Public Health, Department of Social Medicine and Organization of Health Care, Zagreb 10 000, Croatia; obrborov@snz.hr; 2 University of Zagreb, School of Medicine, Andrija Štampar School of Public Health, Department of Environmental and Occupational Health and Sports Medicine, WHO CC for Occupational Health, Zagreb 10 000, Croatia; ianola@snz.hr (I.A.N.); milan.milosevic@snz.hr (M.M.)

**Keywords:** patient safety culture, healthcare workers, healthcare adverse events

## Abstract

*Introduction:* Every procedure in healthcare carries a certain degree of inherent unsafety resulting from problems in practice, which might lead to a healthcare adverse event (HAE). It is very important, and even mandatory, to report HAE. The point of HAE reporting is not to blame the person, but to learn from the HAE in order to prevent future HAEs. *Study question:* Our aim was to examine the prevalence and the impact of culture of blame on health workers’ health. *Methods:* A cross-sectional study on healthcare workers at two Croatian hospitals was conducted using the Hospital Survey on Patient Safety Culture (PSC). *Results*: The majority of PSC dimensions in both hospitals were high. Among the dimensions, Hospital Handoffs and Transitions and Overall Perceptions of Safety had the highest values. The Nonpunitive Response to Error dimension had low values, indicating the ongoing culture of blame. The Staffing dimension had low values, indicating the ongoing shortage of doctors and nurses. *Discussion*: We found inconsistencies between a single-item measure and PSC dimensions. It was expected that Frequency of Events Reported (PSC dimension) relates to Number of Events Reported (single-item measure). However, in our study, the relations between these pairs of measures were different between hospitals. Our results indicate the ongoing culture of blame. Healthcare workers do not report HAE because they fear they will be punished by management or by law.

## 1. Introduction

*Primum non nocere*—patient safety—is a fundamental principle of healthcare [1]. Patient safety represents the prevention of errors and adverse effects to patients associated with healthcare [1,2]. It is an important part of healthcare workers’ everyday work and should be among the professional core values. Every procedure in healthcare carries a certain degree of inherent unsafety resulting from problems in practice, products, procedures, or systems [1]. A healthcare adverse event (HAE) could be any type of error, mistake, incident, accident, or deviation, regardless of whether or not it results in patient harm [3]. Since healthcare is an industry itself, one might assume that the famous Heinrich’s Law on industrial accidents is applicable. Heinrich’s Law states that in a workplace, for every accident that causes a major injury, there are 29 accidents that cause minor injuries and 300 accidents that cause no injuries [4]. Hence, one might assume that for one HAE with a death outcome, there are about 29 HAEs with harm to patients and about 300 HAEs resulting in no injuries. However, this division according to inflicted harm is not recognized worldwide. Moreover, worldwide healthcare lacks valid patient safety measures, since there are so many definitions, regulations, and interpretations on what HAE is and could be [5]. The most cited estimate of annual deaths from medical errors, i.e., HAEs, was reported by Institute of Medicine in 1999. It reported that in the in the United States of America, up to 98,000 deaths are attributed to HAE [6]. However, a recent 2016 report demonstrated even more disturbing and worrying facts. The actual number might be four times higher, resulting in up to 400,000 deaths a year, making HAEs the third most common cause of death [7].

The World Health Organization (WHO) has focused special attention to HAE reporting. It was recognized that similar HAEs or incidents happen to different people under similar circumstances. The point of HAE reporting is not to blame the person, but to learn from the HAE in order to prevent future HAEs. However, the WHO recognized that, despite their mission, there is a global problem of underreporting of incidents and HAEs [2]. Recent data from European Union member states consistently show medical errors and HAEs, with inflicted harm occurring in up to 12% of hospitalizations [1]. For example, in the EU, infections associated with healthcare affect 4.1 million (estimated 1 in 20) hospital patients on average every year [1].

In Croatia, according to the regulations on the quality standards of healthcare and the manner of their application (NN 79/11) [8], hospitals are obliged to submit two types of HAE: 1. Sentinel events reports and 2. Patient safety and staff safety reports to the Agency for Quality and Accreditation in Health Care and Social Welfare. In the last available Agency 2017 report, which comprised reports of 63 Croatian hospitals, there were three types of sentinel events reported and a total of 43 events: Sentinel events related to suicide or attempted suicide (*N* = 42), radiotherapy of the wrong region of the body (*N* = 1), and other types of sentinel events and there were 14 of them (falls in healthcare facility *N* = 8, sudden death after invasive procedure *N* = 1, stillborn *N* = 1, acute lung injury caused by transfusion *N* = 1, death during operating procedure *N* = 1, sudden death *N* = 1, and adverse events during planned embolization *N* = 1). However, there were many more staff safety events reported. A total of 1187 staff safety reports from 63 hospitals were submitted reporting HAEs toward healthcare workers (*N* = 330 physical HAEs, *N* = 720 verbal HAEs, and *N* = 70 material HAEs) [9]. The report showed a global trend of underreporting HAEs despite the courage to report staff safety HAE.

Medical doctors are required to report HAEs. However, there are certain HAEs that nurses can notice and administer/report in their nursing documentation: Falls, decubitus, medicine, and hand hygiene. Other than reporting, both doctors and nurses are required to attend continuous medical education as required by their respective chambers in order to maintain their license. They are also required to follow standards of procedures, checklists (if available and if their departments and colleagues are willing to do so), and guidelines of the best practice. Based on the Law on Quality of Health Care and Social Welfare (NN124/11), hospitals are obliged to constitute a Healthcare Quality Commission [10]. The role of the Commission, which works on the management level, is to ensure that mandatory healthcare quality standards are applied in the hospital. The Commission is also obliged to report to the Agency every six months. The mandatory healthcare quality standards are: Continuous improvement in the quality of clinical and non-clinical procedures (such as systematic review of the use of blood and blood products, antibiotics and medications, systematic review of hospital infections, of the appropriateness of surgical procedures and of medical documentation, waiting time for procedures, duration of hospitalization, time spent in emergency hospital admission, etc.), patient and staff safety, medical documentation, patient rights and experiences, infection control, deaths and autopsy, side effects and adverse events related to medical devices monitoring, internal rating, and supervision of the system and improvement of the quality of healthcare. Despite this being a legal obligation, many hospitals fail to report these mandatory healthcare measure rates, which also raises the question of whether they apply these standards [11].

Although patient safety is difficult to measure, there are questionnaires that measure patient safety culture (PSC), sometimes referred to as patient safety climate [12]. The definition of PSC includes individual and group values, perceptions, competencies, behavioral patterns, and attitudes toward health and safety management in a healthcare organization [13,14,15,16]. PSC is important because patient safety culture (attitudes, beliefs) affects direct actions that comprise patient safety [13]. We might consider patient safety culture (PSC) as a prerequisite for patient safety [13]. Culture is very important, very complex, “contagious and hereditary”, and therefore difficult to change. For example, if a head nurse takes blood without wearing protective gloves, the other nurses might do the same. The other nurses probably will not even question that decision because they “don’t want to get in trouble”. In other words, PSC could be described as “the way we do things around here” [14].

Our previous research, which included a study on medical nurses in one Croatian hospital, showed only two PSC dimensions graded as strong: *Overall Perceptions of Safety* and *Hospital Handoffs & Transitions* [17]. Further research, which included doctors and nurses in two Croatian hospitals, found that nine PSC dimensions were graded strong, but it also found the dimensions *Staffing* and *Nonpunitive Response to Error* to be weak in both hospitals. The *Hospital Management Support for Patient Safety* dimension was found to be weak in one hospital [18].

A larger research, including employees in 10 Croatian hospitals, revealed 11 PSC dimensions to be the strong and only one, *Nonpunitive Response to Error*, to be weak [19]. However, no comparison was done between hospitals.

The primary goal of this study was to examine the prevalence and the impact of culture of blame on health workers’ health.

## 2. Methods

### 2.1. Survey Instrument

The Hospital Survey on PSC questionnaire (HSOPSC) was designed to measure 12 dimensions of PSC [13,15,16]. The HSOPSC questionnaire contained 42 items that mostly use the five-point Likert response scale of agreement (‘Strongly disagree’ to ‘Strongly agree’) or frequency (‘Never’ to ‘Always’). Most of the questionnaire’s items asked respondents to answer using five-point response categories in terms of agreement (‘Strongly agree’, ‘Agree’, ‘Neither’, ‘Disagree’, ‘Strongly disagree’) or frequency (‘Always’, ‘Most of the time’, ‘Sometimes’, ‘Rarely’, ‘Never’). Three of the 12 PSC composites used the frequency response option (‘Feedback and communication about error’, ‘Communication openness’, and ‘Frequency of events reported’), while the other nine composites used the agreement response option. The composite results can be interpreted either as a percentage or a value, where a value of three and lower is weak, three equals neutral, and three and above is strong. The *Safety* grade section requires each participant to assign a grade (on the traditional A to F scale) to patient safety observed by their operational unit (a one-item measure scored from 4 (A) to 0 (F)). A higher score indicated a higher patient safety level. HAE reporting was assessed on a one-item scale that asked, “In the past 12 months, how many adverse event reports have you filled out and submitted?”. Response categories spanned from no adverse event reports (0), 1 to 2 (1), 3 to 5 (2), 6 to 10 (3), 11 to 20 (4), and ≥21 reports (5) [13,15,16]. HAE in the questionnaire was described as any type of error, mistake, incident, accident, or deviation, regardless of whether or not it resulted in patient harm [16]. 

The Croatian translation of the original American HSOPSC showed that 11 dimensions were identified by exploratory factor analysis with acceptable reliability scores compared to the original 12 in the US sample. Five of the twelve dimensions had a Cronbach’s α higher than 0.7, suggesting a reasonable fit to the original HSOPSC. The dimensions *Staffing* and *Organizational learning—Continuous Improvement* were found to have a Cronbach’s α <0.6. The use of confirmatory factor analysis confirmed a good fit to the original American model [13].

### 2.2. Study Procedure

Before the research began, the questionnaire’s items were translated from the original into Croatian by one translator and then translated back into English by an independent, highly qualified medical translator who was blind to the original questionnaires. We employed a cross-sectional study which included doctors and nurses in two Croatian hospitals, Clinical Hospital Centre (CHC) and County General Hospital (CGH). CGH has a bed capacity 369 and 12 departments: Internal medicine, infectiology, dermatology, physical medicine and rehabilitation, neurology, psychiatry, pediatric, surgery, urology, orthopedics, otorhinolaryngology, ophthalmology, gynecology, and anesthesiology. At the time this research took place, there were 104 medical doctors and 221 nurses employed. The average number of patients per year was 17,929 [20].

CHC has a bed capacity of 1207 and is composed of 14 departments: Internal medicine, oncology and radiotherapy, dermatology, physical medicine and rehabilitation, neurology, psychiatry, pediatric, surgery, neurosurgery, urology, otorhinolaryngology, ophthalmology, gynecology, and anesthesiology [20]. 

We decided to approach only doctors and nurses, and excluded other employees, because doctors and nurses are the only ones who can report HAE when it occurs. The research was conducted from January to April 2014. The hospitals were selected using a convenience sample, which involved the employees’ willingness and consent to take part, as well as the geographical proximity to the researchers. In the CHC, 638 doctors and nurses were eligible and willing to participate and 395 participated, comprising a sampling fraction of 61.9%. In CGH, 225 doctors and nurses were eligible to participate and 196 participated, comprising a sampling fraction of 87.1%. The research was anonymous and optional. The questionnaires were distributed in unmarked envelopes along with a consent form. Researchers used morning staff meetings and weekly educational meetings to disseminate the questionnaires personally. The questionnaires were distributed to physicians and nurses who were willing to participate. Following completion, questionnaires and consent forms were returned in separate sealed and unmarked envelopes. The envelopes were then placed by each respondent in a box, which was placed in the nurses’ room in each department. The head nurses of the departments collected the boxes and then returned them to the main investigator [13].

### 2.3. Ethical Permission

Since this research was conducted while the first author was a Biomedicine and Health Studies postgraduate student at the University of Zagreb, School of Medicine, an approval from the School’s ethical board was requested and the study was approved (ref.no. 380-59-10106-19-111/255). Approvals from both hospital board of ethics as well as managers were also requested and obtained (EP-15389/13-10 and 02-7/22-1/2-2011). Participants’ consents were also obtained [13].

### 2.4. Statistical Analysis

All questionnaires were collected and entered into an electronic database and completeness of the data was checked. Since HSOPSC items were worded in both positive and negative directions, negatively worded items first were reverse-coded. The composite scores for PSC were then calculated [13].

All of the continuous data were tested for normality using the Shapiro–Wilk test, and there were no significant departures from normality for all analyzed variables. Categorical data were expressed as frequencies with percentages and continuous data as means with corresponding standard deviations (SD). For all analyses, statistical significance was set at the *p* value of <0.05. Chi-square and Fisher’s exact tests were used for categorical variables. A student t-test and ANOVA were used to analyze differences in continuous variables. 

The analysis was performed using the SAS version 9.1.3 (SAS Institute Inc., SAS Campus Drive, Cary, NC, USA).

## 3. Results

A total of 863 questionnaires were distributed to doctors and nurses present at work at the time the research was conducted, and a total of 591 were returned, comprising a fair response rate of 68.5%, including 150 physicians and 441 nurses. Out of all respondents, 395 (105 physicians and 290 nurses, response rate 61.9%) worked at CHC and 196 (45 physicians and 151 nurses, response rate 87.1%) worked at CGH. The participating physicians and nurses worked in the following departments: Internal medicine (42.4%), surgery (18.8%), anesthesiology and the intensive care unit (ICU) (11.6%), pediatrics (10.1%), obstetrics (9.1%), and psychiatry (8.1%). These departments were included in the standard HSOPSC questionnaire. The aforementioned six departments showed willingness to participate. The internal medicine departments were the biggest in both hospitals, and the largest number of participants who were willing to participate were from this department.

### 3.1. Patient Safety Culture Dimensions

**Figure 1:**
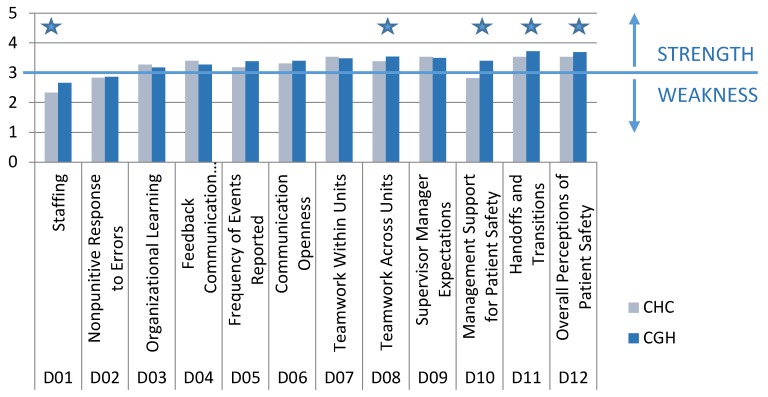
Patient safety culture (PSC) dimensions by hospitals. Pentagram marks statistical difference.

As it can be easily see in Figure 1, the analysis of PSC dimensions revealed that hospitals had many similar cultures. 

Student t-test: Staffing: (t(586) = 5.64, *p* < 0.001), Teamwork Across Hospital Units: (t(588) = 8.28, *p* = 0.005), Hospital Management Support for Patient Safety: (t(585) = 8.28. *p* < 0.001), Hospital Handoffs & Transitions: (t(587) = 3.21. *p* = 0.001), and Overall Perceptions of Safety: (t(589) = 2.84. *p* = 0.005).

Overall, PSC was higher in CGH than in CHC (Figure 1), with eight dimensions scoring higher in CGH. Out of those eight dimensions, a total of five PSC dimensions had statistically significantly higher values:

1. Staffing—X_CHC_ vs. X_CGH_ = 2.33 vs. 2.66, (t(586) = 5.64, *p* < 0.00));
2. Teamwork Across Hospital Units—X_CHC_ vs. X_CGH_ = 3.38 vs. 3.55, (t(588) = 8.28, *p* = 0.005);
3. Hospital Management Support for Patient Safety—X_CHC_ vs. X_CGH_ = 2.81 vs. 3.4 (t(585) = 8.28, *p* < 0.001);
4. Hospital Handoffs & Transitions—X_CHC_ vs. X_CGH_ = 3.53 vs. 3.72 (t(587) = 3.21, *p* = 0.001);
5. *Overall Perceptions of Safety*—X_CHC_ vs. X_CGH_ = 3.53 vs. 3.69, (t(589) = 2.85, *p* = 0.005).

Four PSC dimensions, Organizational Learning, Feedback & Communication About Error, Teamwork Within Hospital Units, and Hospital Management Support for Patient Safety, had higher values in CHC. However, the differences were not statistically significant

Three common characteristics were found for both hospitals:

1. Staffing and Nonpunitive Response to Error were weak in both hospitals;
2. The majority (9/12) of dimensions were strong;
3. Hospital Handoffs and Transitions and Overall Perceptions of Safety had the highest values.

In CHC, Hospital Handoffs and Transitions had the highest values (mean ± SD: 3.80 ± 0.67) (Figure 1). Three dimensions were weak in CHC: Staffing (mean ± SD:2.33 ± 0.62), Nonpunitive Response To Error (mean ± SD:2.84 ± 0.76), and Hospital Management Support for Patient Safety (mean ± SD:2.81 ± 0.79). 

Three dimensions of PSC related to HAE and reporting of HAE showed that:

1. HAE were reported frequently—Frequency of Event Reporting (mean ± SD:3.18 ± 1.19);
2. Healthcare workers were given Feedback and Communication About Error (mean ± SD:3.4 ± 0.81) in a satisfactory manner;
3. Healthcare workers were adequately familiar with Organizational Learning and Continuous Improvement from HAE (mean ± SD:3.27 ± 0.61).

It is interesting that PSC showed that healthcare workers’ perception of managers’ expectations was exceptionally high (Manager expectations and actions promoting safety had values mean ± SD:3.53 ± 0.72). At the same time, they perceived management’s provided support as low (Hospital Management Support for Patient Safety had values mean ± SD:2.81 ± 0.79).

A total of 10 patient safety culture dimensions were strong in CGH (Figure 1). The dimensions Hospital Handoffs & Transitions (mean ± SD:3.72 ± 0.65) and Overall Perceptions of Safety (mean ± SD:3.69 ± 0.68) had the highest values. The dimensions Manager Expectations and Actions Promoting Safety (mean ± SD:3.49 ± 0.86) and Hospital Management Support for Patient Safety (mean ± SD:3.40 ± 0.85) were much more balanced in CGH, and both were strong. The perception of management was that they had high expectations, but also provided the necessary support for patient safety. For the Frequency of Event Reporting dimension, respondents in CGH gave higher grades (X_CHC_ vs. X_CGH_ = 3.18 vs. 3.38).

### 3.2. Number of Healthcare Adverse Events Reported (G1 question)

The vast majority respondents filled out and submitted “no adverse event reports in last 12 months” (in CHC: 70.8%;274/387; in CGH 92.7%: 177/191) (Table 1.). 

In CHC, 113 respondents filled out and submitted reports, while in CGH, statistically significantly less respondents (only 14) did so (Fisher’s exact test: *p* < 0.001). In other words, the majority did report any events (CHC *N* = 274, 70.8%; CGH *N* = 92.7%). We wanted to compare this single-item question with a similarly themed PSC dimension. We expected that they would relate. As seen in the Section 3.1 of the results, the PSC dimension *Frequency of Event Reporting* had a quote good value (XCHC vs. XCGH = 3.18 vs. 3.38), indicating that this dimension was strong. This result suggests that the participants answered that they reported events, which was contradictory to the result of Number of Events Reported question.

### 3.3. Patient Safety Grade (E1 question)

The majority graded the overall grade on patient safety as excellent and very good (Table 2). 

Other grades were acceptable, while only a few responded graded patient safety as poor and only one as failing. The respondents in CGH gave statistically significantly higher grades (Fisher’s exact test: *p* < 0.001). As in the results above, we wanted to investigate whether single-item question and the same themed PSC dimension were related. The *Overall Perceptions of Safety* dimension had higher values in CGH (XCHC vs. XCGH = 3.53 vs. 3.69, (t(589) = 2.85, *p* = 0.005)). We found these two patient safety measures to be consistent. As mentioned, *Patient Safety Grade* is a question for itself, while PSC dimension *Overall Perceptions of Safety* composed four questions (Patient safety is never sacrificed to get more work done (A15); Our procedures and systems are good at preventing errors from happening (A18); It is just by chance that more serious mistakes don’t happen around here (A10 reverse worded); We have patient safety problems in this unit (A17r)).

## 4. Discussion

The analysis of PSC dimensions revealed that hospitals had many similar cultures, with the majority of dimensions graded as strength. The dimensions Hospital Handoffs and Transitions and Overall Perceptions of Safety had the highest values in both hospitals. Five PSC dimensions were statistically significantly higher in CGH: Overall Perceptions of Safety, Staffing, Teamwork Across Hospital Units, Hospital Management Support for Patient Safety and Hospital Handoffs and Transitions. The possible explanation might be that CGH is a smaller hospital where management is not untouchable, but “one of them”. Employed staff stays for the whole career and the turnover rate is low, and the majority of the doctors and nurses know each other well and hence learn to work well in teams. We can also assume that their peer communication (within the department and between departments) is better, as well as within management. Most of our participants were from the internal medicine department (42.35%). These departments were the biggest in both hospitals, employed the largest number of doctors and nurses, and were the most willing to participate.

Weaknesses in both hospitals were found to be Staffing and Nonpunitive Response to Error. The Staffing dimension was not surprising, since it is a known fact that there is a global shortage of healthcare workers [20,21,22]. It was recently announced by the Croatian Medical Chamber that Croatia currently lacks 2125 medical doctors [23].

An inconsistency was found regarding HAE reporting between single-item measure and PSC dimension. It was expected that the Frequency of Events Reported (PSC dimension) would relate with the Number of Events Reported (single-item measure). However, in our study, the relation between this pair was quite different between hospitals. The Number of Events Reported, a single-item measure, revealed that in the past 12 months, 113 respondents in CHC filled out and submitted reports. Meanwhile, in CGH, statistically significantly less respondents (only 14) filled out and submitted reports. However, for the PSC dimension Frequency of Event Reporting, respondents in CGH gave higher grades (XCHC vs. XCGH = 3.18 vs. 3.38). This dimension composed three questions: When a mistake is made but is caught and corrected before affecting the patient, how often is this reported? (D1); When a mistake is made but has no potential to harm the patient, how often is this reported? (D2); When a mistake is made that could harm the patient, but does not, how often is this reported? (D3).

Even though the majority of PSC dimensions were strong in the included hospitals, the finding that the majority did not report any event in the 12-month period still points out that there is a culture of blame present [19,20]. The culture of blame, as described by Peter Pronovost, does not encourage HAE reporting and learning from mistakes, leading to the present fear that “errors shall be punished” [14,24,25]. This result was contradictory to the Values of Dimension Frequency of Event Reporting, which was graded as strong in both hospitals. Another reason for underreporting is the fact that healthcare workers can be punished for their errors with jail time lasting up to 12 years [26]. The Croatian Criminal Law in Head XIX, Article 181, states that doctors or other healthcare workers can be punished with jail time for causing severe harm, worsening of the existing disease or death if it is caused by treatments or procedures that are “obviously inadequate”, malpractice, or if they “obviously fail to comply with the rules of the medical profession” in other ways [26]. There is no definition of “obviously inadequate” in the Criminal Law, so it is left to judge’s interpretation. Meanwhile, the phrase “obviously fail to comply with the rules of the medical profession” is even broader in meaning.

Low values of the *Nonpunitive Response to Error* dimension reflects another global trend. Healthcare workers fear to report HAE and their errors because they fear they will get blamed, punished, and stigmatized [14,24,25]. However, the *Feedback and Communication About Error* dimension was strong in both hospitals. In CGH, PSC was higher than in CHC, but only 14 respondents filled out and reported HAE. It is a concern that HAE reporting is a legal obligation for the hospitals, but the culture (presumably, a culture of blame) in the hospitals is such a strong factor that doctors and nurses sparsely did so. This is a vivid example of how culture can be much more powerful than rules, regulations, and obligations, or as in the popular managerial saying, “culture eats strategy for breakfast” [27]. The official HAE report from 2014 (when this research was conducted) by the Agency for Quality and Accreditation in Health Care and Social Welfare revealed even more troubling results than those in 2017 (mentioned above in the Introduction). Only 49 hospitals (out of 63) delivered full HAE reports to the Agency. Eight types of HAE sentinel events were reported, resulting in 17 HAEs (Instrument or item left at the site of surgery which required additional surgery or additional procedure *N* = 2, transfusion reaction due to AB0 incompatibility *N* = 2, maternal death or severe maternal illness associated with childbirth *N* = 3, the death or permanent disability of a healthy newborn child of greater birth weight of 2500 g, which is not associated with congenital disease *N* = 1, severe neonatal jaundice (bilirubin >513 µmol / L) *N* = 3, radiotherapy of the wrong region of the body *N* = 3, radiotherapy with a dose of 25% above planned *N* = 3), and HAE sentinel events related to suicide or attempted suicide (*N* = 43). These data were not consistent with our findings, which involved two hospitals. However, 76 healthcare facilities (including hospitals) delivered staff safety reports. Staff safety reports from 63 hospitals were submitted, with *N* = 122 physical HAE, *N* = 794 verbal HAE, and *N* = 58 material HAEs reported [28]. 

With two important ideas in mind—that HAE can be any type of error, mistake, incident, accident, or deviation, regardless of whether or not it results in patient harm [3], and that for “every accident that causes a major injury, there are 29 accidents that cause minor injuries and 300 accidents that cause no injuries” (Heinrich’s Law) [4]—it becomes evident that it is beneficial to report every HAE, no matter how small or harmless it might seem, because such a behavior will improve reporting of all types of HAE, even harmful ones.

One of the most important things, which is absolutely valuable for healthcare institution, is to ensure a safe climate for healthcare workers to be honest, come clean, and report the HAE. This way, institutions could create an opportunity for everyone involved in healthcare to learn from others’ HAE and not to repeat the same mistake. Most importantly, in joint efforts, “we allow the system to evolve to create backups that make it easier to detect those mistakes that humans inevitably make” [29]. Three prerequisites are necessary to enable systematic, real-time data on HAE reporting: Valid patient safety measures, timely feedback to clinicians and healthcare organizations, and a positive (blame-free) patient safety culture [5,30].

The questionnaires used in this study was a self-reported questionnaire, which might represent some limitations. Also, the questionnaire used was a few pages long. The literature has shown that when applying long self-reported questionnaires, respondents may find them too long and thus lose interest and not answer the questions accurately [31]. Even though the research was anonymous, individuals may try to hide their true behavior, thoughts, and attitudes [31]. Another limitation is the potential for sample bias due to lack of random selection. Another limitation is that the database is limited and further research with a wider database is warranted.

Patient safety culture values in both hospitals were high. However, important results pointed out what we, as a medical community, still need to work on. First, the vast majority of respondents did not report any HAE in the past. Furthermore, the *Nonpunitive Response to Error* dimension had low values, indicating the ongoing culture of blame. Healthcare workers do not report HAE because they fear they will be punished either by management or by law.

## 5. Conclusions

We believe that the healthcare workers’ behavior described in this study might present a lost opportunity to empower our ancient imperative, *primum non nocere*. Reporting HAE as it happens, thus sharing our experience with other healthcare workers and learning from our mistakes, should become an important part of our professional culture and a core value. It should become the golden rule.

## Figures and Tables

**Table 1:** Number of Events Reported.

Events Reported (*N*)	CHC *	CGH **	TOTAL
	*N* (%)	*N* (%)	*N*
None	274 (70.8)	177 (92.7)	451
1 to 2	40 (10.3)	12 (6.3)	52
3 to 5	30 (7.8)	1 (0.5)	31
6 to 10	26 (6.7)	0 (0.0)	26
11 to 20	8 (2.1)	1 (0.5)	9
21 and more	9 (2.3)	0 (0.0)	9
TOTAL	387	191	578

Fisher’s exact test: *p* < 0.001. * CHC—Clinical Hospital Centre, ** CGH—County General Hospital.

**Table 2:** Patient Safety Grade.

Patient Safety Grade	CHC	CGH	TOTAL
	*N* (%)	*N* (%)	*N*
Excellent	64 (16.2)	63 (33.9)	127
Very Good	183 (46.2)	76 (40.9)	259
Acceptable	139 (35.1)	42 (22.6)	181
Poor	9 (2.3)	5 (2.7)	14
Failing	1 (0.3)	0 (0.0)	1
TOTAL	396	186	582

Fisher’s exact test: *p* < 0,001 CHC—Clinical Hospital Centre, CGH—County General Hospital.
